# Development of independent dose verification plugin using Eclipse scripting API for brachytherapy

**DOI:** 10.1093/jrr/rrac063

**Published:** 2022-10-09

**Authors:** Dejun Zhou, Mitsuhiro Nakamura, Yohei Sawada, Tomohiro Ono, Hideaki Hirashima, Hiraku Iramina, Takanori Adachi, Takahiro Fujimoto, Takashi Mizowaki

**Affiliations:** Department of Information Technology and Medical Engineering, Human Health Sciences, Graduate School of Medicine, Kyoto University, Kyoto 606-8507, Japan; Department of Information Technology and Medical Engineering, Human Health Sciences, Graduate School of Medicine, Kyoto University, Kyoto 606-8507, Japan; Department of Radiation Oncology and Image-Applied Therapy, Graduate School of Medicine, Kyoto University, Kyoto 606-8507, Japan; Division of Clinical Radiology Service, Kyoto University Hospital, Kyoto 606-8507, Japan; Department of Radiation Oncology and Image-Applied Therapy, Graduate School of Medicine, Kyoto University, Kyoto 606-8507, Japan; Department of Radiation Oncology and Image-Applied Therapy, Graduate School of Medicine, Kyoto University, Kyoto 606-8507, Japan; Department of Radiation Oncology and Image-Applied Therapy, Graduate School of Medicine, Kyoto University, Kyoto 606-8507, Japan; Department of Radiation Oncology and Image-Applied Therapy, Graduate School of Medicine, Kyoto University, Kyoto 606-8507, Japan; Division of Clinical Radiology Service, Kyoto University Hospital, Kyoto 606-8507, Japan; Department of Radiation Oncology and Image-Applied Therapy, Graduate School of Medicine, Kyoto University, Kyoto 606-8507, Japan

**Keywords:** brachytherapy, independent dose verification, eclipse scripting

## Abstract

In this study, an independent dose verification plugin (DVP) using the Eclipse Scripting Application Programming Interface (ESAPI) for brachytherapy was developed. The DVP was based on the general 2D formalism reported in AAPM-TG43U1. The coordinate and orientation of each source position were extracted from the translation matrix acquired from the treatment planning system (TPS), and the distance between the source and verification point (*r*) was calculated. Moreover, the angles subtended by the center-tip and tip-tip of the hypothetical line source with respect to the verification point (*θ* and *β*) were calculated. With *r*, *θ*, *β* and the active length of the source acquired from the TPS, the geometry function was calculated. As the TPS calculated the radial dose function, *g*(*r*), and 2D anisotropy function, *F*(*r,θ*), by interpolating and extrapolating the corresponding table stored in the TPS, the DVP calculated *g*(*r*) and *F*(*r,θ*) independently from equations fitted with the Monte Carlo data. The relative deviation of the fitted *g*(*r*) and *F*(*r,θ*) for the GammaMed Plus HDR ^192^Ir source was 0.5% and 0.9%, respectively. The acceptance range of the relative dose difference was set to ±1.03% based on the relative deviation between the fitted functions and Monte Carlo data, and the linear error propagation law. For 64 verification points from sixteen plans, the mean of absolute values of the relative dose difference was 0.19%. The standard deviation (SD) of the relative dose difference was 0.17%. The DVP maximizes efficiency and minimizes human error for the brachytherapy plan check.

## INTRODUCTION

Brachytherapy delivers a high dose to the target volume while realizing a steep dose falling away from the target. Compared with external beam radiotherapy (EBRT), brachytherapy can protect normal tissue while increasing the dose to the target volume. Brachytherapy treatments are conducted with a small fraction and large dose per fraction. Once there is a difference between the planned and delivered doses, it is hard to compensate for the negative effect caused by the difference [1]. Multiple works have recommended performing independent dose verifications to assess the dose calculated by the treatment planning system (TPS) before the brachytherapy treatment [2–4]. Currently the calculation-based verification is the realistic way to check the brachytherapy plan before dose delivery rather than measurement-based verification.

**Fig. 1:**
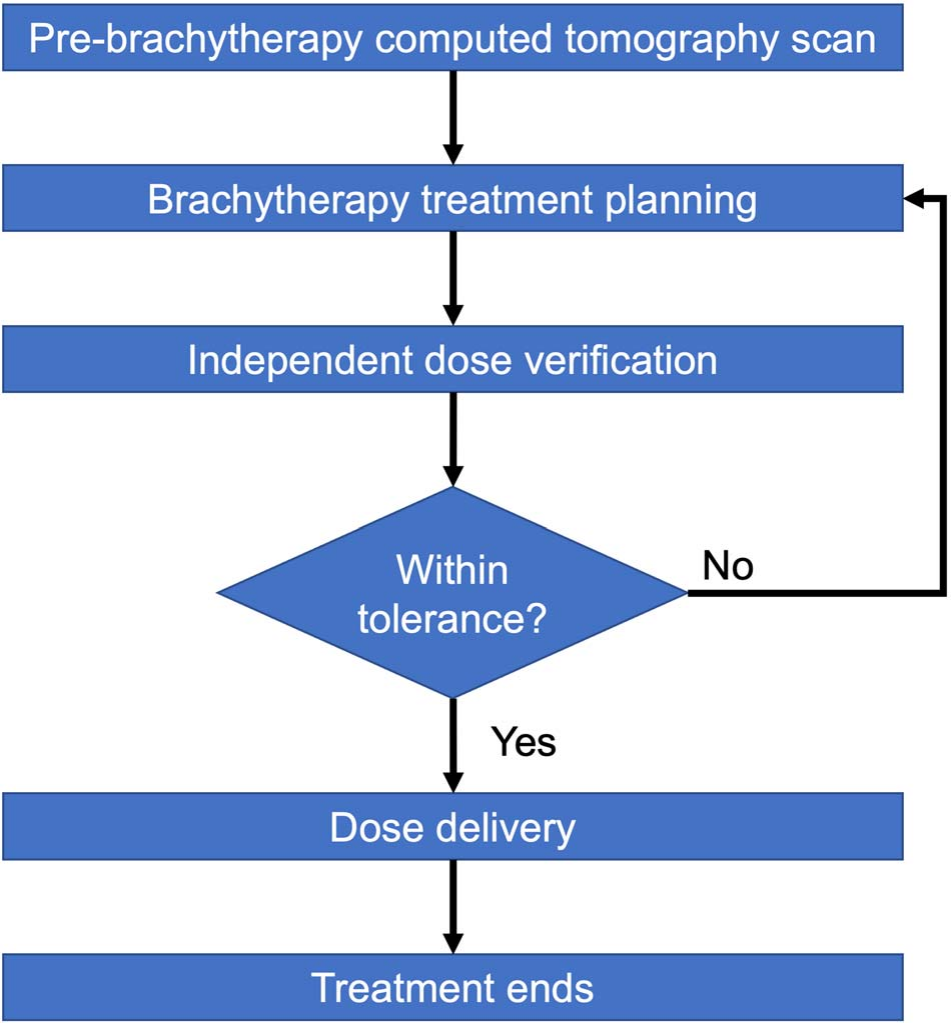
The flowchart of brachytherapy treatment.

The whole process of brachytherapy treatment in our hospital is shown in Fig. 1. First, the patient underwent computed tomography (CT) simulation with applicators. The brachytherapy treatment plan is then made based on the planning CT images. We do not change applicator setup during treatment planning. The independent dose verification is conducted after brachytherapy treatment planning. If the relative dose difference is within the tolerance, the brachytherapy will be conducted and dose will be delivered. If not, the treatment will be re-planned until the verification pass. Currently, in our institution, a Microsoft Excel-based independent dose verification is under clinical practice. Shortcomings of current verification method were revealed. Software other than the TPS was not allowed to be installed on clinical treatment planning machines. To perform the dose verification, the plan information was transferred from the machine with the TPS to another machine with the Microsoft Excel application and pasted to the template file; then, the verification dose was calculated. This procedure is not efficient and may induce human error. Moreover, as the Microsoft Excel-based independent dose verification does not adapt the orientation of each source position in the calculation, the current acceptance range of the relative dose difference of a selected dose verification point is a ± 5% setting between the calculation results of the TPS and verification results. This is a relatively large range compared to the one used in the study reported by Carmona *et al*. [5], where the relative dose difference was within ±2%. A potentially large acceptance range may give a false positive verification judgement.

To overcome the shortcomings, an independent dose verification plugin (DVP) using Eclipse Scripting Application Programming Interface (ESAPI; Varian Medical Systems, Palo Alto, CA, USA) for brachytherapy was developed [6]. The purpose of this study was to simplify the dose verification procedure for brachytherapy and improve the accuracy of the verification dose calculation.

## MATERIALS AND METHODS

### Description of dose verification plugin

In our institution, we use a Bravos unit (Varian Medical Systems) with the GammaMed Plus HDR ^192^Ir active source and BrachyVision V16.1 (Varian Medical Systems) for brachytherapy treatment planning. For this reason, the DVP was dedicated to the Varian TPS and could be integrated with the TPS interface. The DVP was written in C# using ESAPI. With ESAPI, the DVP can access the plan information directly from the TPS and show the dose verification report on the clinical machine with few clicks. There is no need to export, copy and paste data between computers and files. The plan details, especially the orientation of each source position, were adapted in the dose verification calculation in the DVP. In this way, the DVP will show more accurate calculation results, and the acceptance range for the dose verification will be narrowed. The source file of the DVP was one single file. It is easy to update the plugin and distribute it between institutions.

### Dose calculation

The DVP calculates the dose at a verification point (*P_ver_*) independently and compares the TPS results at the same point to verify the dose calculation. The dose calculation algorithm of the DVP was the general 2D formalism reported in the Association of Physicists in Medicine’s update, Task Group 43 (AAPM-TG43U1) [7].

(1) \begin{equation*} \dot{{D}}\left({r},\theta \right)={{S}}_{{k}}\bullet \Lambda \bullet \frac{{{G}}_{{L}}\left({r},\theta \right)}{{{G}}_{{L}}\left({{r}}_0,{\theta}_0\right)}\bullet{{g}}_{{L}}\left({r}\right)\bullet{F}\left({r},\theta \right), \end{equation*}

where *r* is the distance from the center of the active source to *P_ver_*. *θ* is the angle subtended by the central axis of the active source and the line connecting the center of the active source and *P_ver_*. *r*_0_ and *θ*_0_ are specified to 1 cm and 90°, respectively, according to AAPM-TG43U1. *S_k_* is the air-kerma strength on the treatment day. *Λ* is the dose rate constant of the active source. *G_L_* is the geometry function. *g_L_* is the radial dose function, and *F_L_* is the 2D anisotropy function.

The DVP accesses the calibration *S_k_* and half-life of the active source as well as the calibration and treatment dates at 12 a.m. directly from the TPS with ESAPI. Subsequently, the value of *S_k_* at the day of treatment was calculated using the information above and the law of radioactive decay. The DVP also directly acquires Λ from the TPS with ESAPI.

The active source in our institution is a line source. The DVP calculates the *G_L_* based on the line-source model reported in AAPM-TG43U1 [7].

(2) \begin{equation*} {\displaystyle \begin{array}{c}{{G}}_{{L}}\left({r},\theta \right)=\left\{\begin{array}{c}\frac{\beta}{{L}{rsin}\theta},{if}\ \theta \ne 0{}^{\circ}\\{}{\left({{r}}^2-\frac{{{L}}^2}{4}\right)}^{-1},{if}\ \theta =0{}^{\circ}\end{array},\right.\end{array}} \end{equation*}

where *L* is the active length of the source. *β* is the angle subtended by the tips of the hypothetical line source with respect to the *P_ver_*.

The first step of calculating *G_L_* was to obtain the 3D coordinates of the active source center, both active source tips, and *P_ver_*. The active length of the source was stored in the TPS, and the DVP acquired it directly. The TPS stored the position and orientation of the source with a transform matrix. The third column is the source orientation, and the fourth column is the center position. The positions of both tips of the source were calculated by the center position plus/minus the results of half of the active length, multiplied by the source orientation. The position of *P_ver_* was defined in the TPS and directly acquired by the DVP. With the coordinate’s information above, *r*, *θ, β* and *G_L_* were calculated.

**Table 1:** Fitted parameters of the anisotropy function for GammaMed Plus HDR ^192^Ir. Zero values are represented by dashes

*i*	*k_i_*	*a_i_*	*b_i_*	*e_i_*
1	−2.30569	-	4.97$\times$10^−1^	−1.7$\times$10^−3^
2	−1.98$\times$10^−2^	-	−1.46	−2.96
3	2.847$\times$10^−2^	−3.25$\times$10^−1^	5.2$\times$10^−1^	-
4	2.27378	11.5962	24.586	1.469
*i*		${a}_i^{\prime }$	${b}_i^{\prime }$	${e}_i^{\prime }$
1		-	−14.54	−1.14$\times$10^−1^
2		-	−1.5588$\times$10^−1^	−1.057
3		−6.3265$\times$10^−1^	−4.47$\times$10^−1^	−1.81$\times$10^−2^
4		17.0192	39.889	1.2924

In the TPS, *g_L_* and *F_L_* were calculated by interpolating and extrapolating the corresponding data table stored in the radioactive source model. In the DVP, these two functions were calculated independently with fitted dosimetric parameters and equations reported by Lliso *et al*. [8]. The function for *g_L_* was:

(3) \begin{equation*} {\displaystyle \begin{array}{c}{{g}}_{{L}}\left({r}\right)=\frac{{h}{{r}}^{{i}}}{1+{j}{{r}}^{{k}}},\end{array}} \end{equation*}

where for the GammaMed Plus HDR ^192^Ir source, *h*, *i*, *j* and *k* were equals to 1.001, 7.69 $\times$10^−3^, 2.1$\times$10^−4^ and 2.63, respectively.

The general functional forms of *F_L_* were

(4) \begin{equation*} {\displaystyle \begin{array}{c}{F}\left({r},\theta \right)={k}\left({r}\right)+\frac{{a}\left({r}\right){\left(\frac{\theta}{180{}^{\circ}}\right)}^{{e}\left({r}\right)}}{1+{b}\left({r}\right){\left(\frac{\theta}{180{}^{\circ}}\right)}^{{e}\left({r}\right)}}+\frac{{{a}}^{\prime}\left({r}\right){\left(1-\frac{\theta}{180{}^{\circ}}\right)}^{{{e}}^{\prime}\left({r}\right)}}{1+{{b}}^{\prime}\left({r}\right){\left(1-\frac{\theta}{180{}^{\circ}}\right)}^{{{e}}^{\prime}\left({r}\right)}},\end{array}} \end{equation*}

where

$$ {k}\left({r}\right)={{k}}_1{{r}}^{{{k}}_2}+{{k}}_3{r}+{{k}}_4, $$

$$ {a}\left({r}\right)={{a}}_1{{r}}^{{{a}}_2}+{{a}}_3{r}+{{a}}_4,{{a}}^{\prime}\left({r}\right)={{a}}_1^{\prime }{{r}}^{{{a}}_2^{\prime }}+{{a}}_3^{\prime}{r}+{{a}}_4^{\prime } $$

$$ {b}\left({r}\right)={{b}}_1{{r}}^{{{b}}_2}+{{b}}_3{r}+{{b}}_4,{{b}}^{\prime}\left({r}\right)={{b}}_1^{\prime }{{r}}^{{{b}}_2^{\prime }}+{{b}}_3^{\prime}{r}+{{b}}_4^{\prime } $$

$$ {e}\left({r}\right)={{e}}_1{{r}}^{{{e}}_2}+{{e}}_3{r}+{{e}}_4,{{e}}^{\prime}\left({r}\right)={{e}}_1^{\prime }{{r}}^{{{e}}_2^{\prime }}+{{e}}_3^{\prime}{r}+{{e}}_4^{\prime } $$

The fitted parameters of *F_L_* for the GammaMed Plus HDR ^192^Ir are summarized in Table 1**.**

Subsequently, the dose rate of each source position at *P_ver_* was calculated using equation 1, the DVP calculated the dose at *P_ver_,* using the following equation:

(5) \begin{equation*} {\displaystyle \begin{array}{c}{{D}}_{{D}{VP}}=\sum_{{i}=1}^{{N}}\dot{{{D}}_{{i}}}\left({r},\theta \right)\bullet{{t}}_{{i}},\end{array}} \end{equation*}

where *i* is the index of the active source in the plan, and *t* is the dwell time.

### Dose verification

The *P_ver_* dose calculated by the DVP was compared with the dose calculated by the TPS. The relative dose difference *(D_diff%_*) was calculated using the following equation:

(6) \begin{equation*} {\displaystyle \begin{array}{c}{{D}}_{{diff}\%}=100\%\times \frac{{{D}}_{{D}{VP}}-{{D}}_{{TPS}}}{{{D}}_{{TPS}}},\end{array}} \end{equation*}

where *D_TPS_* was the dose at *P_ver_* calculated by the TPS.

**Fig. 2:**
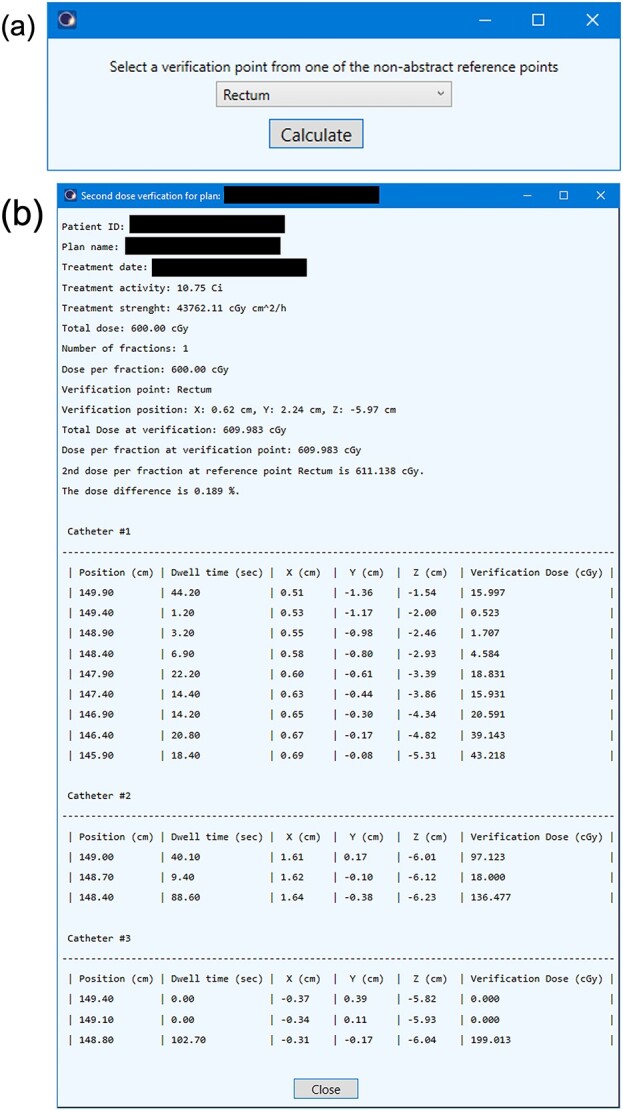
User interface of the DVP. (a) The dose verification point selection window. (b) The dose verification report windows.

According to Lliso *et al*. [8], the average absolute value of the relative deviation between the anisotropy function and Monte Carlo data was 0.9% for the GammaMed Plus HDR ^192^Ir source. For the radial dose function, the value was 0.5%. Based on the linear error propagation theory,

(7) \begin{equation*} {\displaystyle \begin{array}{c}\Delta \left({{g}}_{{L}}\bullet{{F}}_{{L}}\right)=\pm \sqrt{{\left(\Delta{{g}}_{{L}}\right)}^2+{\left(\Delta{{F}}_{{L}}\right)}^2,}\end{array}} \end{equation*}

where $\Delta{g}_L$ and $\Delta{F}_L$ were the average relative deviations of *g_L_* and *F_L_*, respectively. $\Delta \big({g}_L\bullet{F}_L\big)$ was the average relative deviation of *g_L_* multiplied by that of *F_L_*. We assumed that, other than *g_L_* and *F_L_*, there were no deviations in the other components of the dose calculation. In this case, we set ±1.03% as the acceptance range for *D_diff%_*.

### Patient characteristics

This study was approved by the Institutional Review Board of Kyoto University Hospital (approval number: R1446). Our institution started treating patients with the Bravos system from April 2022. Three patients who underwent brachytherapy treatment were included in this study. Two patients were treated with tandem-ovoid applicators, and one was treated with a tandem cylinder. The fractional dose was 6 Gy. At the time of writing, two patients underwent 3 fractions, and one patient underwent 1 fraction. The patients underwent a computer tomography scan and were prepared for each fraction. For each plan, four verification points located at the bladder, rectum, point A at the left side and point A at the right side of the tandem, were determined by the on-site medical physicists or radiation technician, according to the International Commission on Radiation Units and Measurements Report 38 recommendations [9].

### Clinical workflow

The user interface of the DVP is shown in Fig. 2. Before running the DVP, at least one reference point with a location should be selected as the dose verification point and stored in the reference point list of the current plan. The first window of the DVP was the dose verification-point selection window (Fig. 2a). The list of the combo box contained all non-abstract reference points of the plan. After selection and clicking the ‘calculate’ button, the calculation report was shown in the next window (Fig. 2b). The calculation report contains important treatment information, *D_DVP_*, *D*_*TPS*,_*D_diff%_* and the dose calculated by the DVP at each source position.

**Fig. 3:**
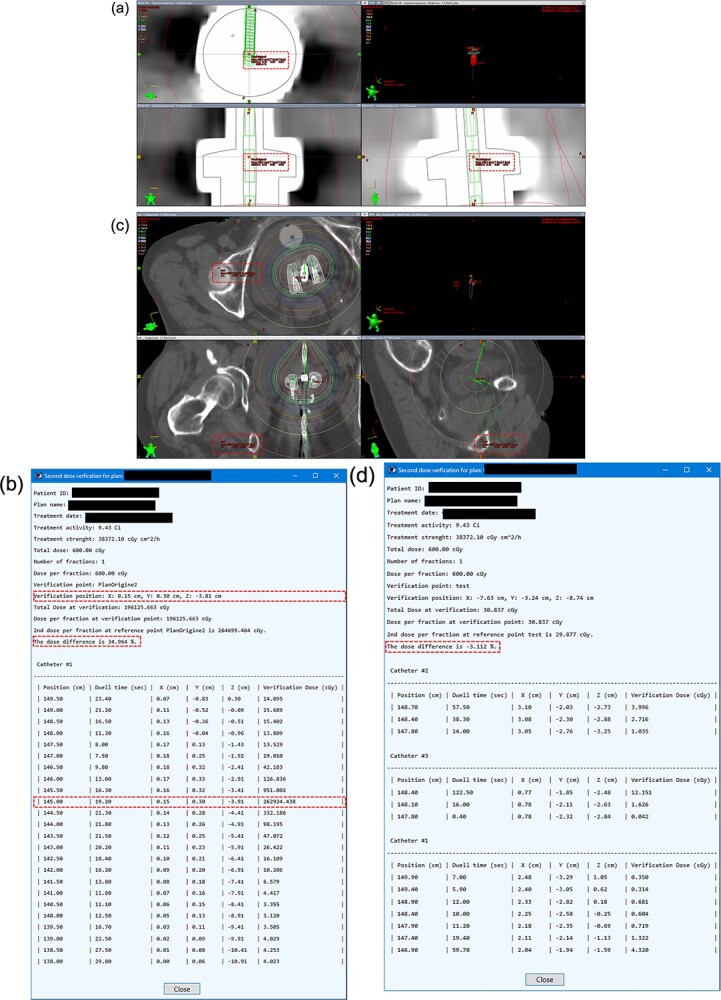
Demonstration of the failed dose verification when the dose verification point was too close to one of the source positions ([a] and [b]), and too far from the sources ([c] and [d]).

## RESULTS AND DISCUSSION

For 16 plans, 64 verification points were selected and included in the statistic results. The mean of the absolute *D_diff%_* was 0.19%. The standard deviation (SD) of *D_diff%_* was 0.17%.

Among all the 64 results, the calculation result of the DVP was smaller than that of the TPS for 83% of verification points (53 verification points). This was caused by the curve of the fitted $\Delta{g}_L$, and $\Delta{F}_L$ was always below the curve of the Monte Carlo simulated data, as reported by Lliso *et al*. [8].

In the two situations, the absolute *D_diff%_* may be out of the acceptance range. First, if the verification point position was inside one of the source positions, a large dose difference was observed. As shown in Fig. 3a–b, the verification point was inside one of the source positions. *D_diff%_* of this verification point was 34.96%.

Second, when the verification point was intentionally positioned far from the sources, *D_diff%_* was out of the acceptance range, as shown in Fig. 3c–d. *D_diff%_* of this verification point was −3.11%. Both situations were caused by the dosimetric parameters used to calculate *g_L_* and *F_L_* in the TPS, and the DVP was not accurate when the verification point was too close or far from the sources. Once *D_diff%_* is greater than the tolerance, the location of the verification point needs to be checked for appropriateness. If the verification point is confirmed as appropriate, the plan may need optimization. In this way, the purpose of the dose verification for brachytherapy is achieved.

This work presented the DVP with GammaMed Plus HDR ^192^Ir source. However, the DVP can be extended to verify brachytherapy with other type of active sources easily. All need to do is to change the fitted parameters of *g_L_* and *F_L_* to the parameters of the corresponding active source according to previous work [8].

## CONCLUSION

An independent DVP dedicated to Eclipse TPS for brachytherapy was developed. For the GammaMed Plus HDR ^192^Ir source, the acceptance range of the relative dose difference between the TPS and plugin was ±1.03%. For 64 verification points, the mean of the absolute values of the relative dose difference was 0.19%. The SD of the relative dose difference was 0.17%. The entire clinical workflow of the plugin contained a few clicks. Once the plugin is under clinical practice, it will maximize efficiency and minimize human error for the brachytherapy plan check before treatment. The code of the DVP will be shared upon reasonable request.

## CONFLICT OF INTEREST

The authors declare they have no conflicts of interest.
